# Supplementary material for: Regulation of EZH2 Expression by INPP4B in Normal Prostate and Primary Prostate Cancer
Source: Cancers (Basel). 2023 Nov 15;15(22):5418. doi: 10.3390/cancers15225418 (PMC10670027; doi:10.3390/cancers15225418)
Supplement: Supplementary file 1 [file cancers-15-05418-s001.zip › 20231023 uncropped blot (2).pptm]

## Slide 1
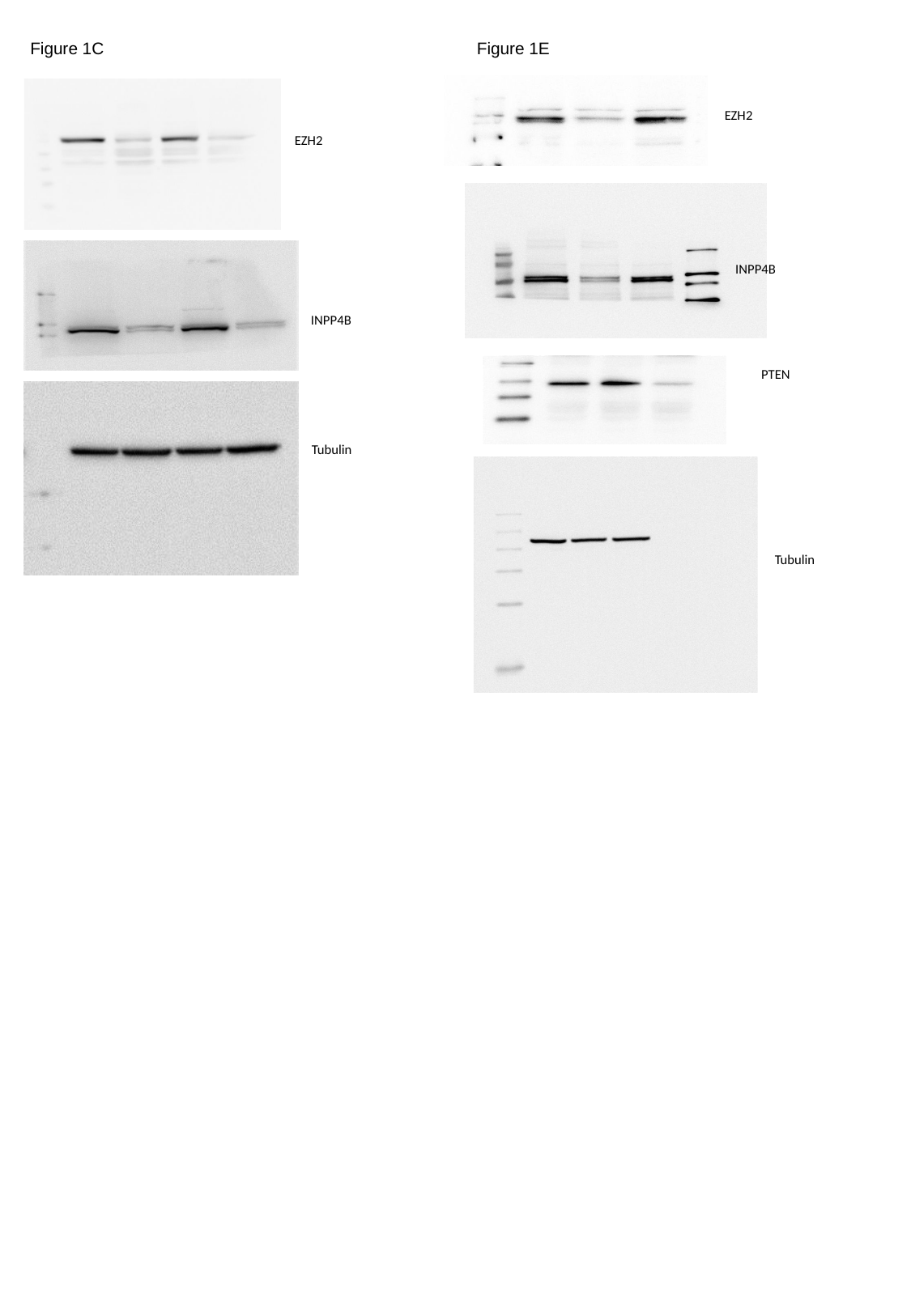

Figure 1E
Figure 1C
EZH2
EZH2
INPP4B
INPP4B
PTEN
Tubulin
Tubulin

## Slide 2
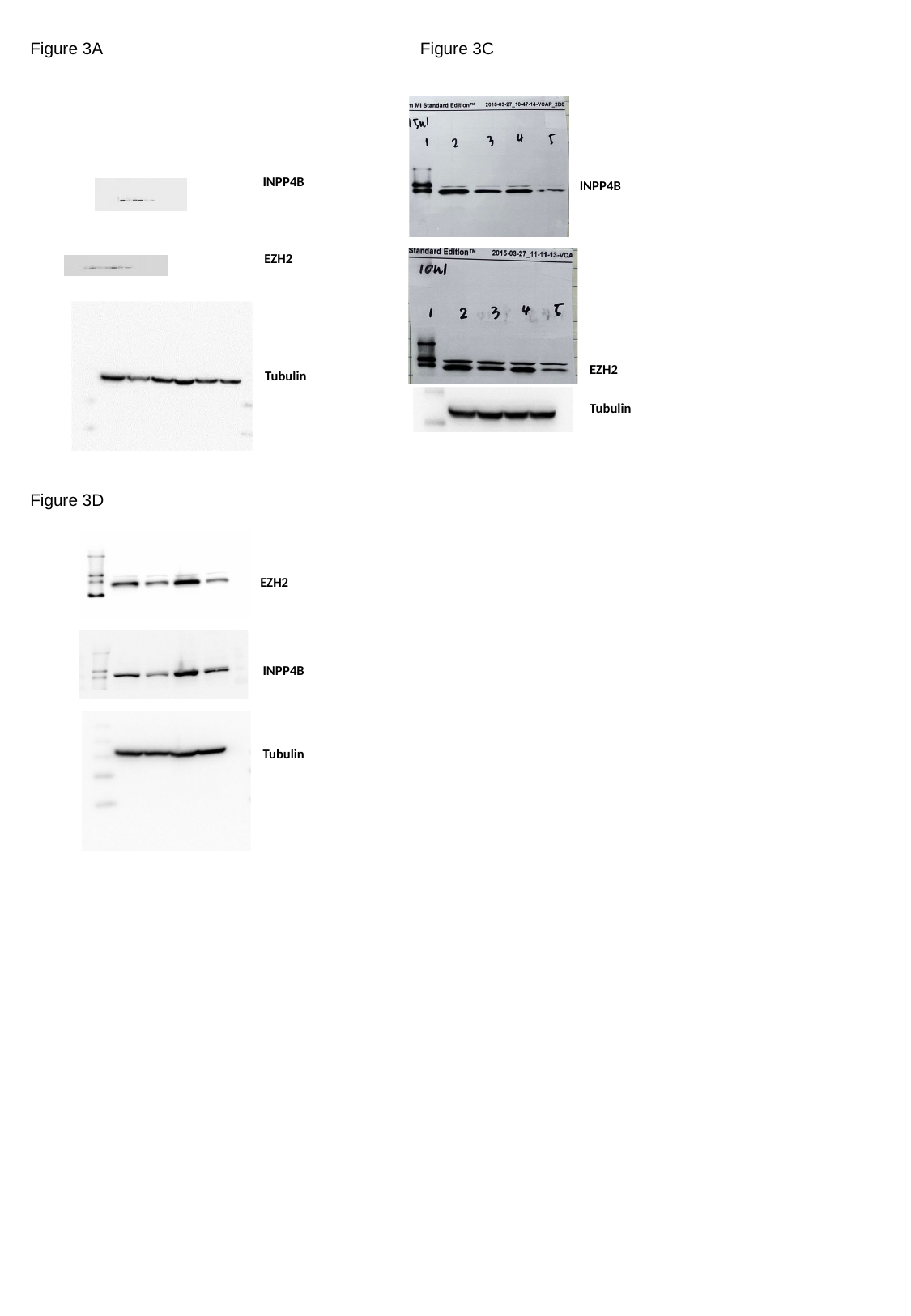

Figure 3A
Figure 3C
INPP4B
INPP4B
EZH2
EZH2
Tubulin
Tubulin
Figure 3D
EZH2
INPP4B
Tubulin

## Slide 3
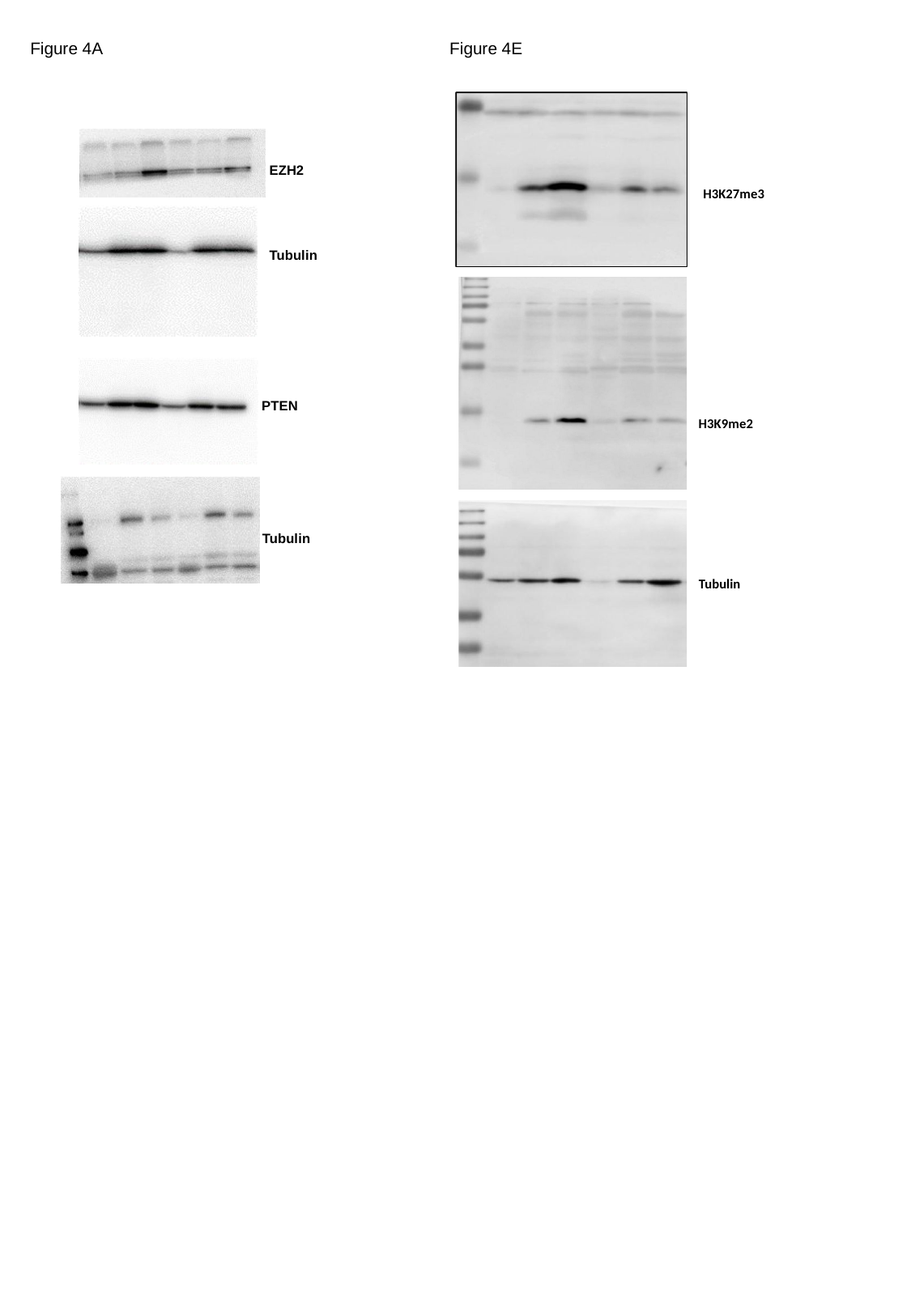

Figure 4A
Figure 4E
EZH2
H3K27me3
Tubulin
PTEN
H3K9me2
Tubulin
Tubulin

## Slide 4
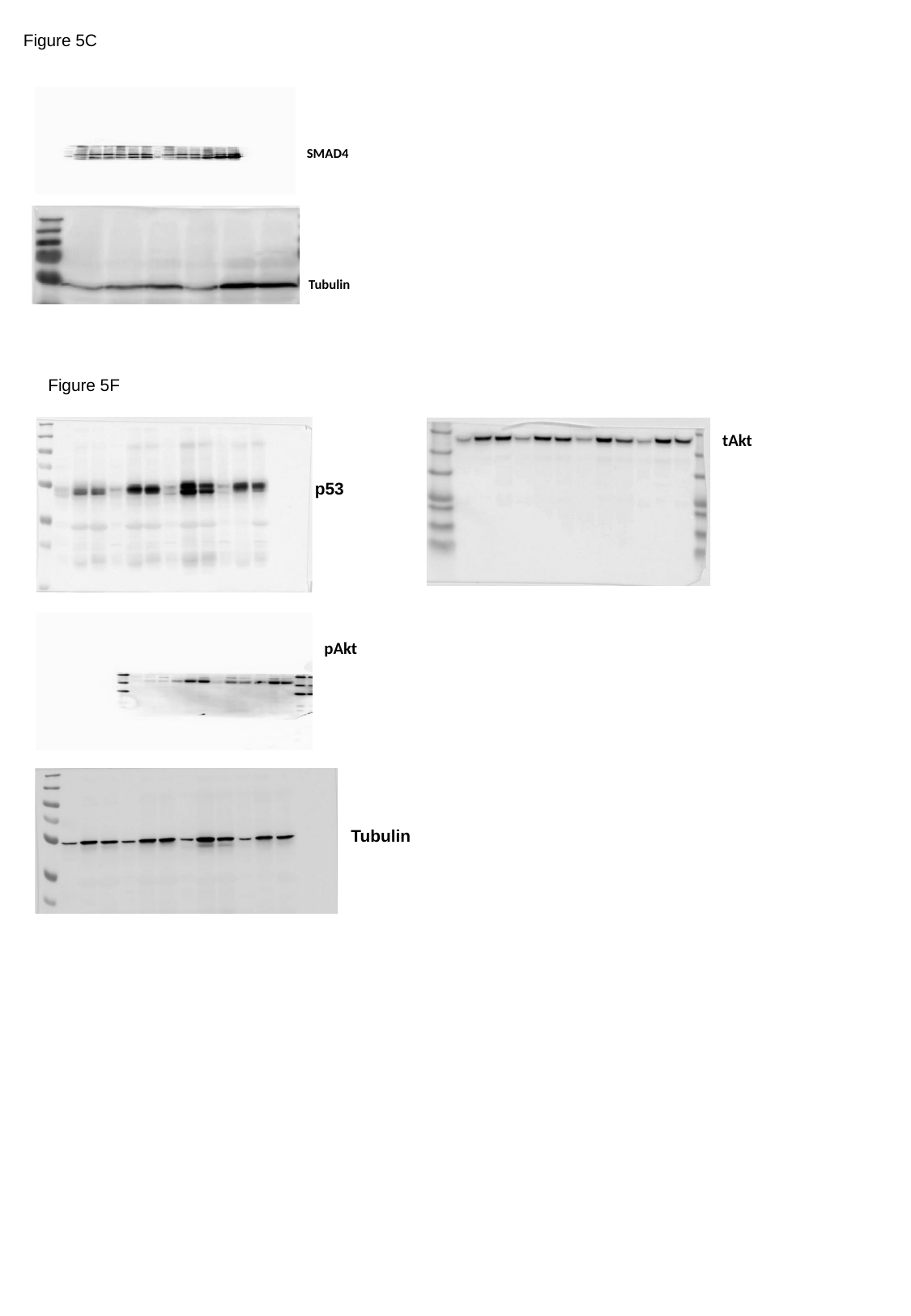

Figure 5C
SMAD4
Tubulin
Figure 5F
tAkt
p53
pAkt
Tubulin
