# Supplementary material for: Regulation of EZH2 Expression by INPP4B in Normal Prostate and Primary Prostate Cancer
Source: Cancers (Basel). 2023 Nov 15;15(22):5418. doi: 10.3390/cancers15225418 (PMC10670027; doi:10.3390/cancers15225418)
Supplement: Supplementary file 1 [file cancers-15-05418-s001.zip › Supplementary figures 20230808.pptx]

## Slide 1
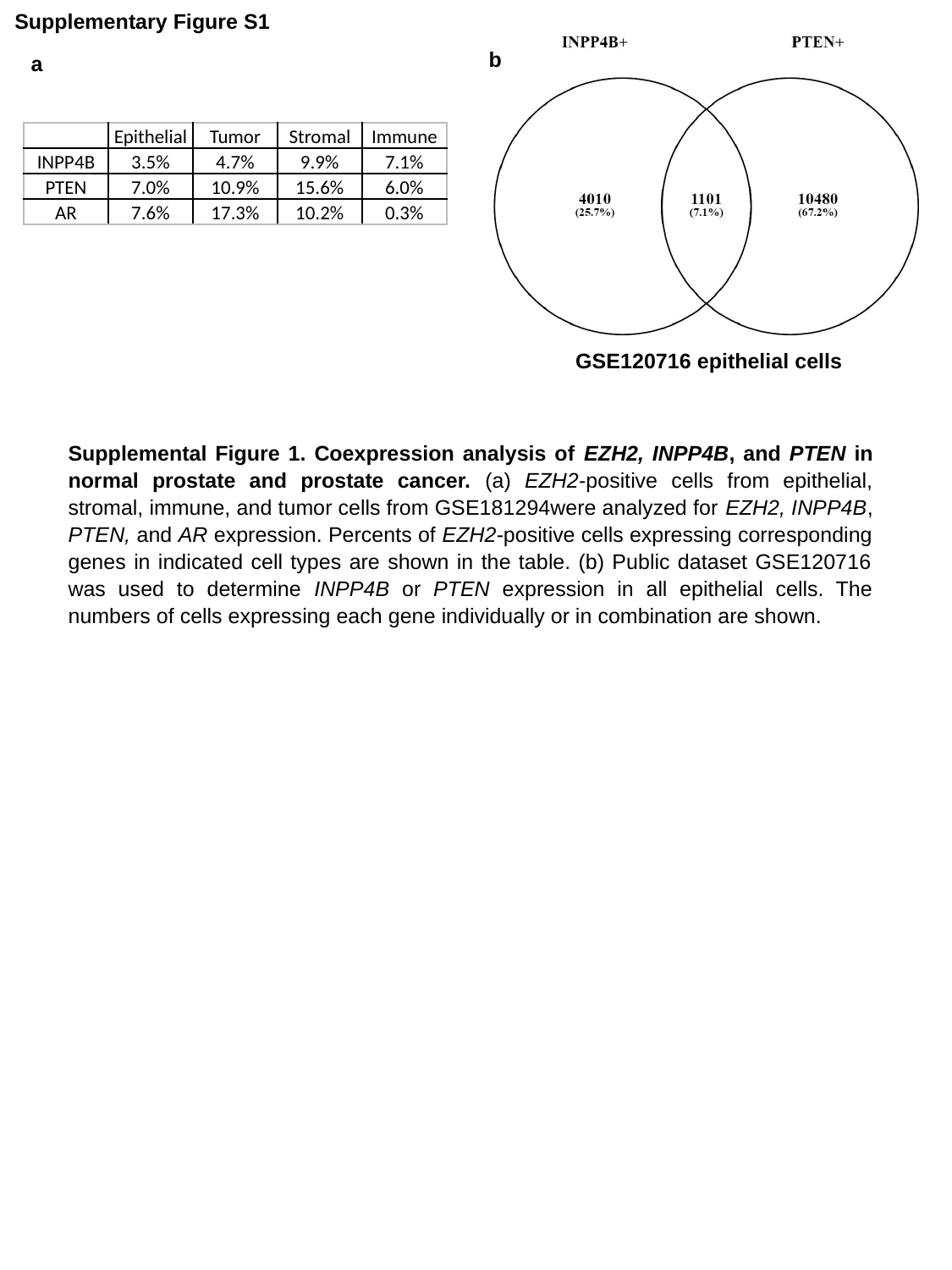

Supplementary Figure S1
b
a
| | Epithelial | Tumor | Stromal | Immune |
| --- | --- | --- | --- | --- |
| INPP4B | 3.5% | 4.7% | 9.9% | 7.1% |
| PTEN | 7.0% | 10.9% | 15.6% | 6.0% |
| AR | 7.6% | 17.3% | 10.2% | 0.3% |
GSE120716 epithelial cells
Supplemental Figure 1. Coexpression analysis of EZH2, INPP4B, and PTEN in normal prostate and prostate cancer. (a) EZH2-positive cells from epithelial, stromal, immune, and tumor cells from GSE181294were analyzed for EZH2, INPP4B, PTEN, and AR expression. Percents of EZH2-positive cells expressing corresponding genes in indicated cell types are shown in the table. (b) Public dataset GSE120716 was used to determine INPP4B or PTEN expression in all epithelial cells. The numbers of cells expressing each gene individually or in combination are shown.
